# The relation between communicative internet usage and the quality of the social adaptation in men with mental disorders

**DOI:** 10.1192/j.eurpsy.2021.2093

**Published:** 2021-08-13

**Authors:** O. Boyko, T. Medvedeva, S. Enikolopov, O. Vorontsova, O. Kazmina, M. Omelchenko

**Affiliations:** Clinical Psychology, Federal State Budgetary Scientific institution “Mental health research center”, Moscow, Russian Federation

**Keywords:** Internet, social adaptation, communication, Mental disorders

## Abstract

**Introduction:**

The internet is now widely used by people with mental disorders, and it is important to understand whether the internet use can affect the mental state of such people.

**Objectives:**

The aim of the study was to assess the relation between the internet usage for communication and the quality of the social adaptation in men with mental disorders.

**Methods:**

82 male patients with schizophrenia spectrum disorder (F20) were involved into the study (mean age 22±4.3). Methods: SCL-90R, “social circle” inventory (Susan L., Phillips), semi-structured interview of “internet usage” (“communicative internet usage” consists of communication in on-line games, use of the internet social networks for communication, use the internet to find new friends, maintaining relationships with relatives, friends, colleagues.

**Results:**

Two groups of patients were considered: those who use internet for communicative purposes (N=61) and those who do not (N=21).According to the analysis (Mann-Witney U-test, hereinafter significance level *p<0.05), those who use the internet for communication have lower levels of psychotic symptoms (PSY) (U=446*), lower levels of “depression” (U=453*). Those who use the internet for communication have more people in social circle to spend free time (U=910,5*), having the same occupation (U=860*), having the same interests (U=867,5*) and sharing the same values (U=873*).They have more friends (U=804*), peers (U=814*), more women among friends (U=793*), more people to provide instrumental support (U=761,5*).

**Conclusions:**

Patients, who use the internet for communications, have a lower levels of psychopathological symptoms and higher quality of social adaptation. This indicates a possible potential of the internet for mental health rehabilitation.

**Disclosure:**

No significant relationships.

